# Social Interaction as a vital factor in alleviating depressive symptoms among middle-aged and elderly adults: evidence from the CHARLS

**DOI:** 10.1007/s40520-025-02941-9

**Published:** 2025-04-07

**Authors:** Chao Wang, Zhipeng Huang, Zuxun Lu, Peigang Wang

**Affiliations:** 1 School of Public Health, Wuhan University, Wuhan, China; 2 School of Public Health, Tongji Medical College, Huazhong University of Science and Technology, Wuhan, China

**Keywords:** Social interaction, Depressive symptoms, The middle-aged and elderly adults, Mental health

## Abstract

**Objective:**

To explore the effect of social interaction on depressive symptoms in middle-aged and elderly adults in China.

**Methods:**

Data from the 2020 China Health and Retirement Longitudinal Study (CHARLS) were analyzed. Participants were divided into social interaction and non-social interaction groups. Depressive symptoms were assessed using the Center for Epidemiologic Studies Depression Scale (CESD-10). Propensity score matching (PSM) was employed to balance confounding factors, and the average treatment effect (ATT) of social interaction on depressive symptoms was estimated. Binary logistic regression analyzed influencing factors.

**Results:**

A total of 14,741 subjects (76.26%) were included; 9,869 (66.91%) participated in social interaction, and 5,593 (37.94%) had depressive symptoms. After PSM, social interaction significantly negatively affected depressive symptoms (ATT=-0.04, *P* < 0.05). Binary logistic regression revealed that those with social interaction had a lower risk of depressive symptoms (OR = 0.87, *P* < 0.05), particularly males (OR = 0.53, *P* < 0.05). However, older age (60–69: OR = 1.20; 70–79: OR = 1.24), poorer self-rated health (general: OR = 2.20; poor: OR = 4.48; very poor: OR = 7.70), lower satisfaction (general: OR = 1.67; dissatisfaction: OR = 8.10), and infrequent meetings with children (every six months: OR = 1.20; less than half a year: OR = 1.27) were associated with a higher risk of depressive symptoms (*P* < 0.05).

**Conclusion:**

Middle-aged and elderly Chinese adults have a high risk of depressive symptoms, and social interaction significantly reduces this risk. Promoting social interaction and mental health initiatives can improve the health of middle-aged and elderly individuals.

## Introduction

Affected by the processes of social and economic development and urbanization [1], youth frequently migrate from their hometowns to regions offering better economic opportunities for employment and settlement, which leads to the hollowing out and estrangement of communities, which in turn contributes to reduced social interaction among the middle-aged and elderly adults, commonly referred to as “empty nesters” [2]. Moreover, owing to the physical frailty often associated with aging, middle-aged and elderly individuals frequently experience a decline in social connections, thereby reducing their participation in social activities [3]. Particularly since the onset of 2020, the COVID-19 pandemic has led many regions to enforce lockdown measures. Epidemic prevention protocols, such as “social distancing” and “not going out unless necessary”, have compelled individuals of all ages to limit or altogether cease social interactions within their homes.

With the advent of the Internet era, online social communication has gradually become an integral part of social interaction [4]. However, middle-aged and elderly individuals often struggle with understanding and navigating internet-based social communication platforms, which makes it difficult for them to fully integrate into online social interactions. Due to the limitation of network and real social interaction activities, emotional support of middle-aged and elderly people will be reduced to a certain extent, and become an important factor affecting their mental health. Depressive symptoms are an important index to evaluate mental health. According to the 2023 research report [5], the detection rate of depressive symptoms in China’s middle-aged and elderly people is 33.10%, which is significantly higher than the 23.2% reported among middle-aged and elderly people in Europe [6] and the 11% reported among middle-aged and elderly people in Singapore [7], indicating that depressive symptoms in China’s middle-aged and elderly population are at a relatively high level. The occurrence of depressive symptoms in middle-aged and elderly people will not only affect their quality of life, daily living ability, but also increase the risk of chronic diseases such as Alzheimer’s disease [8, 9].

Given that the degree of social interaction participation of different populations is limited by the unique economic and social factors of each region, results based on a particular population may not be extrapolated to the population as a whole. In addition, in observational studies, the distribution of some observed variables is uneven among groups, and selection bias is unavoidable due to the “self-selection” of samples [10, 11]. A propensity score (PS) is the probability that an individual will accept a processing effect while controlling for covariates. PSM [12] is the practice of matching individuals with similar propensity scores to reduce confounding bias and selection bias, creating a comparable treatment and control group to more accurately estimate the impact of treatment effects. This method can effectively reduce the bias of observational data, so it is widely used in observational research. Using the data of the China Health and Retirement Longitudinal Study (CHARLS) in 2020, this paper uses PSM to explore the influence of social communication on the occurrence of depressive symptoms in middle-aged and elderly people and its potential mechanism. It is of great significance for clarifying the key entry point of intervention and protecting the mental health of middle-aged and elderly people.

## Information and methods

### Research object

**Fig. 1:**
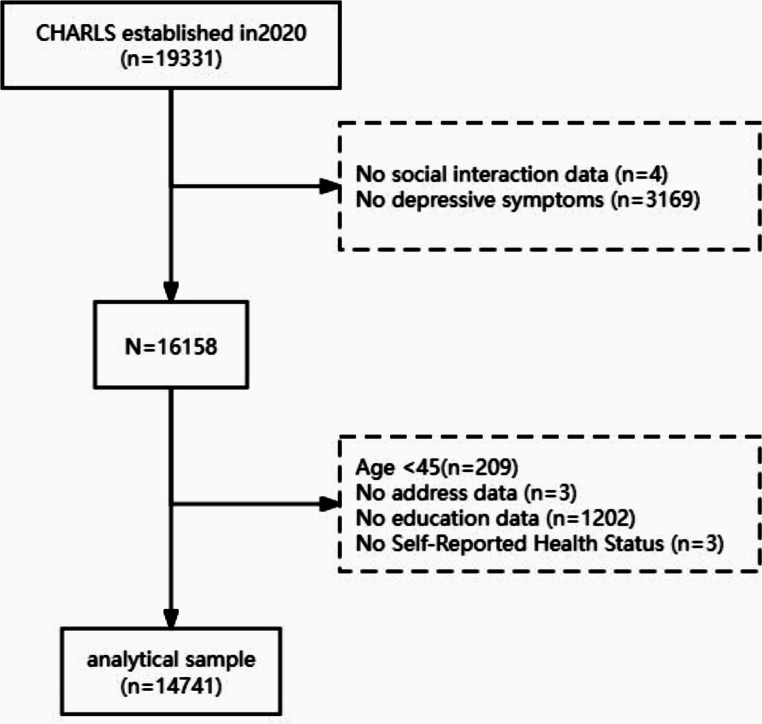
Flow diagram for participants included in the study

The China Health and Retirement Longitudinal Study (CHARLS) aims to collect a high-quality set of micro-level data representative of Chinese households and individuals aged 45 and above. The data is intended to facilitate the analysis of population aging issues in China and promote interdisciplinary research on active aging. The CHARLS national baseline survey was launched in 2011, covering 150 county-level units, 450 village-level units, and nearly 20,000 individuals from approximately 10,000 households. These samples are followed up every two to three years [13]. To date, CHARLS has released five waves of national baseline survey data, conducted in 2011, 2013, 2015, 2018, and 2020. This study utilizes the fifth wave of data collected between June 2021 to August 2023, which includes a total of 19,331 samples. The medical ethics committee approved the CHARLS study, and all interviewees were required to sign informed consent, Ethics approval for the data collection in CHARLS was obtained from the Biomedical Ethics Review Committee of Peking University (IRB00001052–11015). Among them, 19,327 respondents answered the Social Interaction Questionnaire and 16,158 respondents answered the Center for Epidemiological Studies Depression Scale (CESD-10) [14]. Inclusion criteria: (1) at least 45 years old; (2) Complete data from the social interaction questionnaire; (3) Complete CESD-10 scale data; (4) The key variables [Age, Gender, Marital status, Place of residence, Chronic disease, Education level, Self-rated health, Smoking, Drinking, Life satisfaction, Frequency of meetings with children] were not missing. Finally, 14,741 valid samples (76.26%) were included. Please refer to Fig. 1.

### Variable definition

#### Definition of argument variables

In the internet era, significant changes have occurred in people’s social interaction patterns, which are no longer confined to traditional offline methods. Online social activities have become an indispensable part of people’s daily lives due to their convenience and ability to transcend regional boundaries [15]. Therefore, to comprehensively evaluate an individual’s social interaction, this study considers both traditional offline and modern online activities. The questions used to measure social interaction were derived from the Social Interaction Questionnaire within the CHARLS 2020 survey, which includes two key components: (1) “Have you engaged in social activities in the past month?”(e.g., visiting friends, playing Mahjong, going to community activity rooms, participating in club activities) and (2) “Have you gone online in the past month?” (e.g., chatting on the Internet). These questions capture both offline and online social interaction behaviors. If respondents reported participating in any of the eight offline activities listed under the question “Have you engaged in social activities in the past month?” or answered “Yes” to the question about online activity, they were categorized as “participating in social activities” and assigned a value of 1. Conversely, those who did not engage in any of the eight offline activities and did not go online in the past month were categorized as “not participating in social activities” and assigned a value of 0. This classification ensures a comprehensive assessment of social interaction by integrating both offline and online behaviors.

#### Definition of dependent variables

Depressive symptoms were assessed using CESD-10 [16]. There are 10 items in the scale, and each item has four grades (0, 1, 2, 3), which are scored according to the occurrence frequency of events. The higher the frequency of events, the higher the score. The fifth and eighth items are scored in reverse [17]. Subsequently, the total score of the depression scale is obtained by summing up the ratings of all 10 items, ranging from 0 to 30, and the higher the score, the more serious the depression symptoms of the individual. This scale has been widely used in several studies [18, 19] and has been well validated in the measurement of depression in middle-aged and elderly people. The Cronbach’s coefficient of this scale is 0.82, which has good reliability and validity [16]. Referring to a number of previous studies [20, 21], the total score of the scale ≥ 10 indicates depressive symptoms, and is defined as 1; A total score of < 10 on the scale indicates no symptoms of depression and is defined as 0.

#### Definition of covariables

With reference to the research of Wang Yue [22], demographic variables (age, gender and marriage), socioeconomic variables (place of residence and education level), health status variables (chronic disease and self-rated health), behavioral lifestyle (smoking, drinking), quality of life variables (life satisfaction) and family relationship variables (Frequency of meetings with children) were included as possible confounding variables. Marriage refers to the current marital status, divided into married and non-married; Chronic diseases are diagnosed by doctors; Residence refers to the current residential area, divided into Urban community and Rural village; Education level refers to the highest educational attainment of the respondents, which is divided into primary education and below, secondary education level and higher education level; Smoking means still smoking, non-smoking means quit smoking and never smoking; Drinking refers to consuming alcohol more than once a month and less than once a month in the past year, while not drinking refers to never consuming alcohol in the past year; Life satisfaction is divided into “very satisfied”, “somewhat satisfied”, and “dissatisfied”; The number of times children see each other refers to how often they can see their children when they are not living together. The assignment of variables is shown in Table 1.

### Statistical methods

Stata17.0 software was utilized for conducting statistical analysis. Categorical variables were described using frequencies and percentages, while the *X*^2^ test was employed to compare between groups. Binary logistic regression was applied to analyze the impact of social interaction on depressive symptoms in middle-aged and elderly individuals. Additionally, PSM of a counterfactual research framework was conducted as follows: (1) PS was calculated based on a logit model, and covariates were matched using K-nearest neighbor matching with a caliper of k = 4, radius matching with a radius of 0.01, and nuclear matching with default kernel and bandwidth settings to match the treatment group (social interaction group) with the control group (non-social interaction group), aiming to assess the effects of social interaction on depressive symptoms in middle-aged and elderly individuals; (2) The standardized difference (STD) was computed to assess the covariate distribution balance between the two groups, with previous studies [23, 24] indicating that an STD value below 10% is indicative of a relatively balanced variable distribution; (3) The construction of a deviation reduction chart was undertaken to conduct a common support test; (4) The results obtained from the matched control group served as counterfactual outcomes for the treatment group, enabling calculation of differences in detection rates of depressive symptoms between the matched treatment and control groups as an estimation of social interaction’s influence on depressive symptoms—referred to as average treatment effect on treated (ATT). The ATT symbol signifies both directionality and magnitude regarding how social interaction affects depressive symptoms [25]. Statistical significance level adopted throughout analyses is *P* < 0.05.

## Results

### General information

The average age of the participants was (60.71 ± 9.11) years. There were 7082 male participants(48.04%), 7659 female participants(51.96%); 5150 participants (34.94%) residing in urban community, 9591 participants (65.06%) residing in rural village; 12,680 married participants(86.02%),and 2061 non-married participants (13.98%). The detailed information is presented in Table 1.

**Table 1:** Social interaction and depressive symptoms detection of middle-aged and elderly people under different characteristic variables

Variables		*n*(%)	Social interaction		X^2^	*P*	Depressive symptoms detection		X^2^	*P*
			Yes(*n*%)	No(*n*%)			Yes(*n*%)	No(*n*%)		
**Age(years)**					1100	< 0.001			101.41	< 0.001
45–59	1	6973(47.30)	5559(79.72)	1414(20.28)			2363(33.89)	4610(66.11)		
60–69	2	5152(34.95)	3044(59.08)	2108(40.92)			2085(40.47)	3067(59.73)		
70–79	3	2216(15.03)	1087(49.05)	1129(50.95)			979(44.18)	1237(55.82)		
80-	4	400(2.71)	173(43.25)	227(56.75)			166(41.50)	234(58.50)		
**Gender**					4.50	0.03			430.94	< 0.001
Male	1	7082(48.04)	4799(67.76)	2283(32.24)			2076(29.31)	5006(70.69)		
Female	0	7659(51.96)	5064(66.12)	2595(33.88)			3517(45.92)	4142(54.08)		
**Address**					316.02	< 0.001			227.86	< 0.001
Urban community	1	5150(34.94)	3930(76.31)	1220(23.69)			1530(29.71)	3620(70.29)		
Rural village	0	9591(65.06)	5933(61.86)	3658(38.14)			4063(42.36)	5528(57.64)		
**Marital status**					76.24	< 0.001			163.21	< 0.001
Married	1	12,680(86.02)	8657(68.27)	4023(31.73)			4550(35.88)	8130(64.12)		
Non-married	0	2061(13.98)	1206(58.52)	855(41.48)			1043(50.61)	1018(49.39)		
**Education level**					824.43	< 0.001			459.11	< 0.001
Primary education and below	1	9307(63.14)	5446(58.52)	3861(41.48)			4131(44.39)	5176(55.61)		
Secondary education	2	5140(34.87)	4142(80.58)	998(19.42)			1413(27.49)	3727(72.51)		
Higher education	3	294(1.99)	275(93.54)	19(6.46)			49(16.67)	245(83.33)		
**Self-rated health**					112.20	< 0.001			1700	< 0.001
Good	1	3652(24.77)	2558(70.04)	1094(29.96)			631(17.28)	3021(82.72)		
Fair	2	7513(50.97)	5156(68.63)	2357(31.37)			2716(36.15)	4797(63.85)		
Poor	3	2600(17.64)	1604(61.69)	996(38.31)			1521(58.50)	1079(41.50)		
Very poor	4	976(6.62)	545(55.84)	431(44.16)			725(74.28)	251(25.72)		
**Chronic**					0.00	0.95			171.37	< 0.001
Yes	1	5176(35.11)	3465(66.94)	1711(33.06)			2332(45.05)	2844(54.95)		
No	0	9565(64.89)	6398(66.89)	3167(33.11)			3261(34.09)	6304(65.91)		
**Life satisfaction**					65.33	< 0.001			1400	< 0.001
Very satisfied	1	5260(35.68)	3378(64.22)	1882(35.78)			1401(26.63)	3859(73.37)		
Somewhat satistied	2	7943(53.88)	5538(69.72)	2405(30.28)			2966(37.34)	4977(62.66)		
Dissatisfied	3	1538(10.43)	947(61.57)	591(38.43)			1226(79.71)	312(20.29)		
**Smoking**					11.49	0.001			91.74	< 0.001
Yes	1	3881(26.33)	2682(69.11)	1199(30.89)			1224(31.54)	2657(68.46)		
No	0	10,860(73.67)	7181(66.12)	3679(33.88)			4369(40.23)	6491(59.77)		
**Drinking**					230.23	< 0.001			239.82	< 0.001
Yes	1	5526(37.49)	4117(74.50)	1400(25.50)			1655(29.95)	3871(70.05)		
No	0	9215(62.51)	5746(62.35)	3469(37.65)			3938(42.73)	5277(57.27)		
**Frequency of meetings with children**					37.99	< 0.001			53.07	< 0.001
Once every two weeks or more	1	3971(26.94)	2660(66.99)	1311(33.01)			1326(33.39)	2645(66.61)		
Once every six months	2	4153(28.17)	2924(70.41)	1229(29.59)			1509(38.26)	2564(61.74)		
Less than once every six months	3	6617(44.89)	4279(64.67)	2338(35.33)			2678(40.47)	3939(59.53)		
Total		14,741	9863(66.91)	4878(33.09)			5593(37.94)	9148(62.06)		

### Current situation of social interaction

The results showed that 9255 people (66.45%) participated in social interaction, 4672 people (33.55%) did not participate in social interaction. In social interaction, the proportion of men (67.76%) was higher than that of women (66.12%), that of urban community (76.31%) was higher than that of rural village (61.86%), that of married (68.27%) was higher than that of non-married (58.52%), that of smokers (69.11%) was higher than that of non-smokers (66.12%). Drinking (74.50%) was higher than non-drinking (62.35%), and the differences were statistically significant (*P* < 0.05). The social interaction rates of different age groups, education level, self-rated health, life satisfaction and children meeting times were different, and the differences were statistically significant (*P* < 0.05). Further details are provided in Table 1.

### Prevalence of depressive symptoms

Among the participants, 5593 individuals (37.94%) had depressive symptoms and 9148 individuals (62.06%) had no depressive symptoms. The prevalence of depressive symptoms was higher among women (45.92%) compared to men (29.31%), higher in rural areas (42.36%) than urban areas (29.71%), higher among non-married individuals (50.61%) than married individuals (35.88%), higher among those with chronic diseases (45.05%) than those without chronic diseases (34.09%), higher among non-smokers (40.23%) than smokers (31.54%), and higher among non-drinkers (42.73%) than drinkers (29.95%), and the differences were statistically significant (*P* < 0.05). There were statistically significant differences in social interaction rates across different age groups, education levels, self-rated health, life satisfaction, and frequency of meetings with children (*P* < 0.05). These results are presented in Table 1.

### PSM result

When examining the impact of social interaction on depressive symptoms among middle-aged and elderly individuals, the results revealed significant imbalances in the distribution of covariates. such as age, gender, place of residence, marital status, education level, self-rated health, life satisfaction, smoking, drinking, and frequency of meetings with children between the social interaction group and the non-social interaction group (*P* < 0.05). These imbalances may lead to confounding effects, thereby affecting the accuracy of the conclusions. Although chronic diseases did not show significant imbalances in distribution between the social interaction and non-social interaction groups, existing studies [26, 27] have demonstrated that chronic diseases significantly influence the mental health and depressive symptoms of middle-aged and elderly individuals. Therefore, to comprehensively control for potential confounders and more accurately assess the impact of social interaction on depressive symptoms in middle-aged and elderly individuals, we decided to include the variable of chronic diseases in the PSM analysis.

#### Propensity score estimation

A logistic regression model was constructed with social interaction as the dependent variable. The model included demographic variables (age, gender, and marital status), socioeconomic variables (place of residence and education level), health status variables (presence of chronic diseases and self-rated health), behavioral and lifestyle variables (smoking and alcohol consumption), quality of life variables (life satisfaction), and family relationship variables (frequency of meetings with children) as independent variables. The estimated probability of social interaction among middle-aged and elderly individuals is represented by PS. Refer to Table 2 for details.

**Table 2:** Estimates of social interaction tendency score using logit model

Social interaction	Coefficient	SE	Z	*P*
Age	-0.60	0.02	-24.54	< 0.01
Gender	-0.27	0.05	-5.68	< 0.01
Address	0.46	0.04	10.77	< 0.01
Marital status	-0.07	0.06	-1.35	0.08
Education level	0.81	0.04	18.93	< 0.01
Self-rated health	-0.09	0.02	-3.87	< 0.01
Chronic	0.13	0.04	3.32	< 0.01
Life satisfaction	0.01	0.03	0.19	0.72
Drinking	0.49	0.04	11.01	< 0.01
Smoking	0.11	0.05	2.13	0.06
Frequency of meetings with children	-0.07	0.02	-3.17	< 0.01

#### Test of balance and co-support

Taking K-nearest neighbor matching with calipers as an example, all absolute values of the STD between the matched social interaction group and the non-social interaction group were found to be less than 10%. This indicates a satisfactory comparison between the two groups after undergoing PSM. In order to ensure the matching quality, PSM needs to meet the common support test, that is, it requires the social interaction group and the non-social interaction group to have a large overlap in the value range of propensity scores, otherwise it will easily lead to bias. Among the total 14,741 observed values, 1 of the non-social interaction group was not in the common value range, 136 of the social interaction group was not in the common value range, and the other 14,604 observed values were in the common value range. These results demonstrate the robustness of the study following matching. Please refer to Tables 3 and 4 for further details.

**Table 3:** Reduction of covariate deviation

Variable	Unmatched	Mean		%bias	%reduct	t-test	
	Matched	Treated	Control		|bias|	t	*p*>|t|
Age	U	1.58	2.03	-56.70		-33.01	0.00
	M	1.59	1.59	-0.40	99.30	-0.28	0.78
Gender	U	0.49	0.47	3.70		2.12	0.03
	M	0.48	0.49	-0.70	80.60	-0.50	0.62
Address	U	0.40	0.25	32.10		17.97	0.00
	M	0.39	0.39	-0.20	99.40	-0.13	0.90
Marital status	U	0.88	0.82	14.90		8.75	0.00
	M	0.88	0.87	1.70	88.90	1.23	0.22
Education level	U	1.48	1.21	53.70		29.38	0.00
	M	1.45	1.47	-2.40	95.50	-1.54	0.12
Self-rated health	U	2.01	2.16	-17.10		-9.89	0.00
	M	2.02	2.00	2.50	85.60	1.81	0.07
Chronic	U	0.35	0.35	0.10		0.07	0.95
	M	0.35	0.34	3.30	-2736.80	2.31	0.02
Life satisfaction	U	1.75	1.74	2.80		1.65	0.10
	M	1.75	1.76	-1.10	62.70	-0.77	0.44
Drinking	U	0.42	0.29	27.10		15.29	0.00
	M	0.41	0.40	2.10	92.20	1.42	0.16
Smoking	U	0.27	0.25	6.00		3.39	0.00
	M	0.27	0.26	3.40	43.70	2.32	0.02
frequency of meetings with children	U	2.16	2.21	-5.60		-3.20	0.00
	M	2.16	2.18	-1.70	69.00	-1.21	0.23

**Table 4:** Common support

Treatment assignment	Off support	On support	Total
Untreated	1	4877	4878
Treated	136	9727	9863
Total	137	14,604	14,741

#### The impact of social interaction on depressive symptoms in middle-aged and elderly individuals

The results of t value and ATT value obtained by the three matching methods are basically consistent, which indicates that the results of this study have strong stability. The results showed that the social interaction obtained under different matching methods had statistical significance on the ATT of depressive symptoms in middle-aged and elderly people (ATT=-0.04, *p* < 0.05). Please refer to Table 5 for further details.

**Table 5:** ATT estimates of the association effect between social interaction and depression in middle-aged and elderly people

Matching mode	Treated	Untreated	ATT	SE	T-stat	*P*
K-nearest neighbor matching	0.35	0.39	-0.04	0.01	-3.05	< 0.05
Radius matching	0.35	0.39	-0.04	0.01	-3.67	< 0.05
Kernel matching	0.35	0.39	-0.04	0.01	-3.67	< 0.05

After excluding 137 observations that fell outside the common value range, we applied a binary logistic regression model to examine the impact of independent variables, such as social interaction, on depression symptoms in middle-aged and elderly individuals, with depression symptoms as the dependent variable. Model 1 suggests that engaging in social communication can reduce the risk of depressive symptoms [OR = 0.67, *P* < 0.001]. In Model 2, which included the social interaction variable as well as demographic variables, the study found that the risk of depressive symptoms in the social interaction group was lower than in the non-social interaction group [OR = 0.73, *P* < 0.001]. Additionally, individuals aged 60–69 and 70–79 had a higher risk of depressive symptoms compared to those aged 45–59, with [OR = 1.25, *P* < 0.001] and [OR = 1.35, *P* < 0.001], respectively. Furthermore, the risk of depressive symptoms was higher in females than in males [OR = 2.01, *P* < 0.001], while married middle-aged and elderly individuals had a lower risk of depressive symptoms compared to unmarried individuals [OR = 0.67, *P* < 0.001].In Model 3, which included social interaction, demographic, and socioeconomic variables, the results showed that the risk of depressive symptoms in the social interaction group was still lower than in the non-social interaction group [OR = 0.84, *P* < 0.001]. Individuals aged 60–69 and 70–79 continued to show a higher risk of depressive symptoms compared to those aged 45–59, with [OR = 1.18, *P* < 0.001] and [OR = 1.24, *P* < 0.001], respectively. Similarly, females had a higher risk of depressive symptoms than males [OR = 1.88, *P* < 0.001], while married individuals exhibited a lower risk than unmarried individuals [OR = 0.67, *P* < 0.001]. Moreover, individuals living in urban areas had a lower risk of depressive symptoms compared to those in rural areas [OR = 0.65, *P* < 0.001]. Individuals with secondary education and higher education had a lower risk of depressive symptoms compared to those with primary education or below, with [OR = 0.65, *P* < 0.001] and [OR = 0.44, *P* < 0.001], respectively. In Model 4, which included social interaction, demographic, socioeconomic, and behavioral lifestyle variables, the study found that the risk of depressive symptoms in the social interaction group remained lower than in the non-social interaction group [OR = 0.85, *P* < 0.001]. Individuals aged 60–69 and 70–79 still exhibited a higher risk of depressive symptoms compared to those aged 45–59, with [OR = 1.17, *P* < 0.001] and [OR = 1.23, *P* < 0.001], respectively. Females continued to show a higher risk than males [OR = 1.77, *P* < 0.001], while married individuals had a lower risk than unmarried individuals [OR = 0.67, *P* < 0.001]. Urban residents had a lower risk of depressive symptoms compared to rural residents [OR = 0.65, *P* < 0.001]. Individuals with secondary and higher education showed a lower risk of depressive symptoms compared to those with primary education or below, with [OR = 0.65, *P* < 0.001] and [OR = 0.45, *P* < 0.001], respectively. Additionally, middle-aged and elderly individuals who consumed alcohol had a lower risk of depressive symptoms compared to non-drinkers [OR = 0.81, *P* < 0.001].In Model 5, which included all variables in the analysis, the results showed that the risk of depressive symptoms in the social interaction group remained lower than in the non-social interaction group [OR = 0.87, *P* < 0.01]. Moreover, model 5 reveals that individuals aged 60–69 and 70–79 face a higher risk of depressive symptoms compared to those aged 45–59 [OR = 1.20, *P* < 0.001] and [OR = 1.24, *P* < 0.01], respectively. Additionally, males exhibit a lower risk of depressive symptoms than females [OR = 0.53, *P* < 0.001]. Urban residents also experience a lower risk of depressive symptoms than rural residents [OR = 0.68, *P* < 0.001], while married individuals demonstrate a lower risk than unmarried individuals [OR = 0.71, *P* < 0.001]. Furthermore, the likelihood of experiencing depressive symptoms decreases with higher levels of education, with secondary and higher education levels associated with lower risks than primary education [OR = 0.66, *P* < 0.001], [OR = 0.50, *P* < 0.001], respectively. Conversely, individuals with poorer self-rated health exhibit higher risks of depressive symptoms compared to those with better self-rated health [OR = 2.20, *P* < 0.001], [OR = 4.48, *P* < 0.001], [OR = 7.70, *P* < 0.001]. Moreover, individuals with chronic diseases face higher risks of depressive symptoms than those without [OR = 1.18, *P* < 0.001]. Dissatisfaction with overall satisfaction levels also correlates with higher risks of depressive symptoms compared to high levels of satisfaction [OR = 1.67, *P* < 0.001], [OR = 8.10, *P* < 0.001]. Interestingly, alcohol consumption is associated with a lower risk of depressive symptoms compared to abstinence [OR = 0.90, *P* < 0.05]. Lastly, individuals who see their children semiannually or less frequently have a higher risk of depressive symptoms compared to those who see them once every two weeks [OR = 1.20, *P* < 0.001], [OR = 1.27, *P* < 0.001].Please refer to Table 6 for further details.

**Table 6:** Influencing factors of depressive symptoms in middle-aged and elderly people under multiple models

Variable		Model 1		Model 2		Model 3		Model 4		Model 5	
		OR	95CI%	OR	95CI%	OR	95CI%	OR	95CI%	OR	95CI%
Social interaction	No										
	Yes	0.67***	(0.67,0.72)	0.73***	(0.67,0.78)	0.84***	(0.78,0.90)	0.85***	(0.79,0.92)	0.87**	(0.80,0.95)
Age	45–59										
	60–69			1.25***	(0.16,1.35)	1.18***	(1.09,1.28)	1.17***	(1.08,1.27)	1.20***	(1.10,1.31)
	70–79			1.35***	(1.22,1.51)	1.24***	(1.12,1.38)	1.23***	(1.10,1.38)	1.24**	(1.10,1.40)
	80-			1.06	(0.86,1.33)	0.99	(0.80,1.24)	0.97	(0.78,1.21)	1.17	(0.92,1.49)
Gender	male										
	female			2.01***	(1.88,2.16)	1.88***	(1.75,2.02)	1.77***	(1.63,1.94)	1.90***	(1.72,2.10)
Marital status	Non-married										
	married			0.67***	(0.60,0.74)	0.67***	(0.60,0.74)	0.67***	(0.61,0.74)	0.71***	(0.63,0.79)
Address	rural										
	urban					0.65***	(0.60,0.70)	0.65***	(0.60,0.70)	0.68***	(0.62,0.74)
Education level	Primary education and below										
	Secondary education					0.65***	(0.60,0.70)	0.65***	(0.60,0.71)	0.66***	(0.60,0.72)
	Higher education					0.44***	(0.32,0.60)	0.45***	(0.33,0.62)	0.50***	(0.36,0.70)
Smoking	No										
	Yes							1.05	(0.96,1.15)	1.09	(0.98,1.21)
Drinking	No										
	Yes							0.81***	(0.75,0.89)	0.90*	(0.83,0.99)
Chronic	No										
	Yes									1.18***	(1.09,1.28)
Self-rated health	Good										
	Fair									2.20***	(1.99,2.45)
	Poor									4.48***	(3.95,5.08)
	Very poor									7.70***	(6.42,9.23)
frequency of meetings with children	Once every two weeks or more										
	Once every six months									1.20**	(1.08,1.33)
	Less than once every six months									1.27***	(1.15,1.39)
Life satisfaction	Very satisfied										
	Somewhat satisfied									1.67***	(1.54,1.82)
	dissatisfied									8.10***	(6.97,9.41)

**Model 1** includes demographic variables (age, gender, and marital status); **Model 2** includes demographic variables (age, gender, and marital status)); Model 3:includes demographic variables (age, gender, and marital status), socioeconomic variables (place of residence and education level);**Model 4**:includes demographic variables (age, gender, and marital status), socioeconomic variables (place of residence and education level) and behavioral and lifestyle variables (smoking and alcohol consumption); **Model 5**:includes all variables. *p* < 0.001,***;*p* < 0.01,**;*p* < 0.05,*

## Discussion

Based on CHARLS2020 data, this study explored the association between social interaction and depressive symptoms in middle-aged and elderly people. The study found that the participation of middle-aged and elderly people in social interaction can reduce the occurrence of depressive symptoms. This may be related to the fact that social interaction can provide people with more social support and intimacy. When people are connected to those around them and feel cared for and supported by others, they are more likely to form a positive psychological state [28], thus reducing the risk of depression. In addition, social communication is also an important way for people to realize self-worth, recognition of others and enhance self-confidence. When people succeed in social interactions or receive positive feedback from others, it helps to increase their self-esteem and self-confidence, which reduces the occurrence of depressive symptoms [29]. On the other hand, social interaction also provides more opportunities and resources for middle-aged and elderly people. For example, when communicating and cooperating with others, people can acquire new knowledge, new skills and new life manifestations, which not only helps to improve the quality of life of individuals, but also helps to form positive life attitudes and ways to cope with pressure, and then reduce the negative emotions of middle-aged and elderly people, thus reducing the risk of depression symptoms in middle-aged and elderly people [30]. Therefore, we should strengthen community construction [31], enhance community cohesion, and organize various activities suitable for the middle-aged and elderly, such as square dancing, calligraphy and leisure running.

In terms of geographical distribution, the risk of depressive symptoms is higher in rural areas than in urban areas [32, 33], which may be because social networks in rural areas are different from those in urban areas, and people are more likely to rely on family and village groups for help and support. However, with the development of urbanization and the increase of population flow, the number of residents in rural areas has decreased and cohesion has weakened. Meanwhile, due to the lower economic level in rural areas and the lack of professional mental health services and treatment compared with urban areas, these factors may lead to the increased risk of depressive symptoms in middle-aged and elderly people in rural areas [34]. Therefore, in order to improve mental health in rural areas, it is necessary for the government and society to increase investment in mental health in rural areas and strengthen the construction of community support network.

In the realm of physiology, this study found that women had a higher risk of depressive symptoms than men, which was consistent with the results of Joan Domenech-Abella’s study [35]. This may be due to the fact that women need to go through multiple special life cycles such as menstruation, pregnancy and menopause, and the changes in hormone levels in the body lead to mood swings and depressive symptoms [36]. At the same time, women are more sensitive than men and tend to internalize emotions, which leads to the accumulation of emotions and the occurrence of depression [37]. Moreover, the role requirements and expectations of men and women are different in society, and women often assume family responsibilities such as taking care of families, children and elders, thus reducing social interaction activities with the outside world. These pressures make women feel cumbersome, disappointed and helpless in daily life, and thus increase the risk of depression [38, 39]. Therefore, attention should be paid to providing corresponding social support and resources for women, and increasing psychological monitoring for women in special periods, so as to help women better cope with emotional problems and depressive symptoms [40].

Regarding health factors, people with chronic diseases have a higher risk of depressive symptoms [26], which may be hampered by prolonged chronic diseases, which have adverse psychological effects on them, and then lead to the occurrence of depressive symptoms. Self-rated health is a widely used comprehensive subjective health evaluation index, so the better the self-rated health, the lower the risk of depressive symptoms. In terms of lifestyle, it is worth noting that the incidence of depression in non-smokers and non-drinkers is higher than that in smokers and non-drinkers. However, binary logistic regression analysis found that smoking has no statistical significance, while drinking is a protective factor for depression in middle-aged and elderly people, which is consistent with the research results of Xing Xianjin [41]. This may be because appropriate drinking can play a role in pleasing mood and promoting sleep to a certain extent [42].

Interestingly, this study found that middle-aged and elderly individuals with fewer in-person meetings with their children have a higher risk of developing depressive symptoms. This highlights the significant role of face-to-face parent-child interactions in the mental health of middle-aged and elderly people. For these individuals, their children are one of the most important sources of emotional support [2]. In-person meetings not only provide direct emotional care but also alleviate loneliness and psychological stress through face-to-face communication, thereby reducing the risk of depressive symptoms. Such interactions allow children to better observe their parents’ health conditions, emotional fluctuations, and challenges in daily life, enabling timely identification and intervention in potential problems. However, when the frequency of in-person meetings is low, this critical support is significantly diminished. Moreover, the presence of children can indirectly encourage middle-aged and elderly individuals to participate in more social activities, expanding their social support networks and improving their mental health on multiple levels [43]. Therefore, it is essential for family members to pay greater attention to parent-child interactions in middle-aged and elderly individuals. By promoting family support and emotional interaction, the quality of life and mental health of middle-aged and elderly individuals can be improved, thus reducing the occurrence of depressive symptoms.

To sum up, the current situation of depression in middle-aged and elderly people in China is not optimistic, and social communication can reduce the risk of depressive symptoms. Therefore, the government and relevant departments need to increase the construction of community and rural infrastructure to create a good place for promoting social communication between middle-aged and elderly people. At the same time, focus on women, unmarried, suffering from chronic diseases and low education level of people with depression, strengthen health publicity and education and mental health intervention; In addition, we should strengthen investment in unbalanced development areas such as rural areas, formulate relevant safeguard measures, and promote the equalization of basic public health services, so as to improve the mental health of middle-aged and elderly people, and then improve the quality of life and sense of gain of middle-aged and elderly people.

There are some limitations in this study: (1) The CESD-10 scale used by CHARLS to assess depression symptoms in middle-aged and elderly people is a self-report, which may have recall bias; (2) There are many factors affecting depression in middle-aged and elderly people, and this study may not cover all the influencing factors; (3) Although this study used a large sample of data from a reliable database, it excluded subjects that did not meet the criteria, which affected the extrapolation of the results to some extent.

## Data Availability

The datasets used in this study are available from the China Health and Retirement Longitudinal Study (CHARLS) at https://charls.pku.edu.cn/. These data were obtained with the approval of the CHARLS project team and are not publicly available. For academic use of raw data, please log in to https://charls.pku.edu.cn/ to apply for access. For processed data, please send an email to wu_wangch@163.com. Requests will be evaluated based on institutional and departmental policies to determine whether the requested data are subject to intellectual property or patient privacy obligations. Data can only be shared for non-commercial academic purposes and will require a formal data use agreement.
